# The relationship between the atherogenic index of plasma and arterial stiffness in essential hypertensive patients from China: a cross-sectional study

**DOI:** 10.1186/s12872-021-02049-8

**Published:** 2021-05-19

**Authors:** Juan Yin, Minghui Li, Lingling Yu, Feng Hu, Yu Yu, Longlong Hu, Huihui Bao, Xiaoshu Cheng

**Affiliations:** 1 Department of Cardiology, The Second Affiliated Hospital of Nanchang University, 1 Minde Road, Nanchang, 330006 China; 2 Department of Gerontology, Jiangxi Provincial People’s Hospital Affiliated to Nanchang University, Nanchang, China; 3 Department of Rehabilitation, The Second Affiliated Hospital of Nanchang University, Nanchang, China

**Keywords:** Atherogenic index of plasma, Brachial-ankle pulse wave velocity, Arterial stiffness, Hypertension

## Abstract

**Background:**

The atherogenic index of plasma (AIP) always remains in a potential association with arterial stiffness, however, this association has not been fully discovered and needs to be studied in depth in large hypertensive patient populations. The present analysis thus sought to further explore the association that exists between AIP and arterial stiffness in Chinese patients diagnosed with arterial hypertension.

**Methods:**

This cross-sectional study analyzed 4744 Chinese individuals with essential hypertension. AIP was defined as the base 10 logarithm of the ratio of plasma of triglycerides to high-density lipoprotein cholesterol levels indicated in molar concentrations. Measurement of arterial stiffness was carried out via brachial-ankle pulse wave velocity (baPWV).

**Results:**

Data were adjusted for potential confounding variables, and multivariate linear regression analysis revealed AIP to be positively correlated with baPWV (β = 1.34, 95% CI: 0.96 to 1.72, P < 0.001). When AIP was instead treated as a categorical variable divided into quartiles, the same relationship was observed (P for trend < 0.001). We additionally found AIP and baPWV had a stronger positive association in individuals with a body mass index (BMI) < 24 kg/m^2^ (P for interaction < 0.05).

**Conclusion:**

AIP and arterial stiffness were positively correlated in essential hypertension patients in China, especially in those with a BMI < 24 kg/m^2^.

*Clinical trial registration* ChiCTR1800017274.

## Background

Hypertension has been identified as a risk factor associated with high rates of mortality. About 23.2% of adults in China are estimated to suffer from hypertension, which is only controlled in 15.3% of cases [1]. In individuals suffering from hypertension, arterial stiffness appears to be a reliable prognosticator of subclinical vascular disorders and cardiovascular mortalities [2]. Similarly, the increased stiffness is also indicative of asymptomatic target organ damage in these patients [3]. Increased arterial stiffness has been reported to be associated with elevated cardiovascular risk [4], and thus, represents a valuable metric to guide the stratification of hypertensive patients based on their cardiovascular risk [5].

Carotid-femoral pulse wave velocity (cfPWV) is presently considered as the gold standard method for assessing arterial stiffness [6], whereas brachial-ankle pulse wave velocity (baPWV) is similarly validated as a cardiovascular risk marker and is also closely correlated with aortic PWV and cfPWV [7]. As such, BaPWV has been utilized as a valid, reproducible, and routine tool for the non-invasive assessment of patients in clinical studies [8, 9]. As such, BaPWV was used as a metric for arterial stiffness in the present analysis.

The atherogenic index of plasma (AIP) is an easily calculated index value that is based upon circulating lipid levels in a given patient [10]. Relative to individual lipid parameters, AIP is generally superior as it is more strongly correlated with the distribution of small dense low-density lipoprotein (sdLDL) particles [11], which exhibit more pronounced atherogenic activity than the low- density lipoprotein cholesterol (LDL-c) particles [12]. Many studies have shown AIP to be a predictor of serious cardiovascular incidents and atherosclerosis in individuals with coronary arterial disease [13, 14], type 2 diabetes mellitus [15, 16] and metabolic syndrome [17], as well as in patients undergoing maintenance hemodialysis [18]. While AIP is known to be closely associated with atherosclerosis, its relationship with arterial stiffness is not yet well defined. Indeed, only a few studies to date have assessed the association between AIP and arterial stiffness in hypertensive patients. Choudhary et al. identified that AIP was directly and independently correlated with PWV in 249 normotensive and 366 untreated hypertensive subjects [19]. while Si et al. found a possible correlation between AIP and baPWV in hypertensive patients when analyzing 380 Chinese patients [20].

This study was designed for evaluating the association between AIP and arterial stiffness in a large real-world population of hypertensive Chinese patients.

## Methods

### Participants

All subjects in the present study were participants in an H-type hypertension Registry Study (http://www.chictr.org.cn/searchproj.aspx Registration number: ChiCTR1800017274) conducted in China from March 2018 to August 2018 in Wuyuan, Jiangxi Province, China. The established standards of inclusion or exclusion as well as the data collection approaches related to this study have been described previously [21]. Briefly, this was a real-world observational study of adults over 18 years of age with hypertension as defined by diastolic blood pressure (DBP) ≥ 90 mmHg and/or systolic blood pressure (SBP) ≥ 140 mmHg, or by the use of antihypertensive agents.

**Fig. 1:**
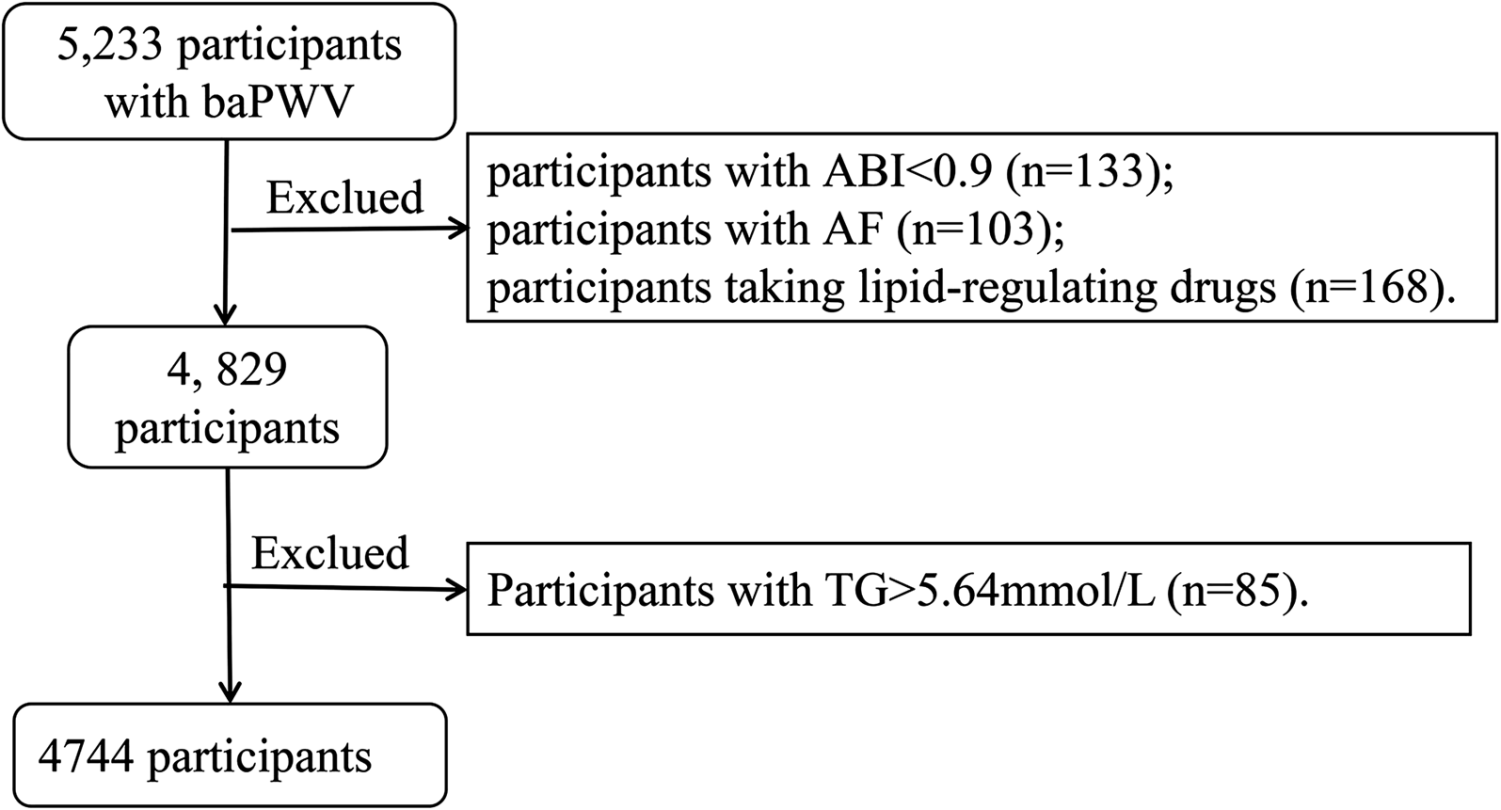
Data flow chart of participants in our analysis. baPWV, brachial-ankle pulse wave velocity; ABI, ankle brachial index; AF, atrial fibrillation; TG, triglycerides

Of the eligible patients, 5233 completed baPWV measurements and were enrolled in the current study. However, 133 patients with ABI < 0.9 were excluded from the study [22], 103 individuals with atrial fibrillation [22], 168 patients taking lipid-regulating medications, and 85 individuals with triglyceride levels ≥ 500 mg/dl [23]. In total, 4744 individuals were considered eligible for the final assessment (Fig. 1). The study was approved by the Ethics Committee of the Institute of Biomedicine, Anhui Medical University, and was also consistent with the Declaration of Helsinki. Patients participating in this study provided their written informed consent.

### Clinical data collection

For each patient, demographic characteristics including age, sex, lifestyle (smoking status, drinking status, and labor intensity), medical history (including atrial fibrillation [AF], stroke, diabetes mellitus, and coronary artery disease), and medication usage (including antihypertensive, lipid-lowering, antiplatelet agents, and hypoglycemic) were recorded. AF was diagnosed based on a medical history and through resting supine standard 12-lead surface electrocardiograms (25 mm/s, 10 mm/mV).

Anthropometric measurements for each participant including weight, waist circumference, SBP, DBP, and heart rate (HR) were obtained by researchers. A validated non-invasive electronic oscillometric device (Omron; Dalian, China) with an appropriate cuff size for the upper arm was used to take four consecutive BP measurements (with a time interval of 1–2 min), with the mean values from the three final recordings being used for analytical purposes to decrease the impact of reactivity on BP. BMI was determined as follows: BMI = weight (kg) /height^2^ (m^2^). Diagnosis of incident diabetes was defined as fasting glucose > 7.0 mmol/l, and/or self-reported diabetes during the follow-up period. All research staff involved in this study underwent identical training to ensure consistency.

### Laboratory assay

Venous blood specimens were obtained from all patients following a 12 h minimum overnight fasting and were stored at 4 ℃. Plasma total homocysteine, low-density lipoprotein cholesterol (LDL-C), total triglyceride, fasting blood glucose, high-density lipoprotein cholesterol (HDL-C), total cholesterol (TC), serum uric acid serum creatinine, and blood urea nitrogen levels were quantified with automated clinical analyzers (Beckman Coulter) at the core laboratory of the National Clinical Research Center for Kidney Disease, Guangzhou, China. The entire laboratory’s measurements were obtained in a manner consistent with a standardization and certification program. The estimated glomerular filtration rate (eGFR) was established with the equation of Chronic Kidney Disease Epidemiology Collaboration (CKD-EPI), as in those with higher eGFR levels this equation exhibits superior accuracy as compared to the equation of Modification of Diet in Renal Disease (MDRD) [24]. AIP was calculated as follows: log_10_ [triglyceride/ HDL-c] with each concentration being presented as mmol/L.

### Measurement of baPWV

BaPWV and the ankle-brachial index (ABI) were measured on the day of blood sample collection using a BP-203RPEIII networked arteriosclerosis detection instrument (Omron Health Care, Kyoto, Japan). Measurements were conducted in a noiseless room with subjects in the supine position following at least 5 min of rest. Participants were not permitted to consume tea, alcohol, coffee, or cigarettes for 30 min prior to testing. The measurement approaches of baPWV and ABI related to this study have been described previously [25]. Four cufs were wrapped around ankles and bilateral brachia followed by their connection to an oscillometric pressure sensor and plethysmographic sensor. The ABI was measured by dividing ankle SBP divided with the brachial SBP. Recording of the pressure waveforms was carried out with semiconductor pressure sensors for assessing the transmission time between the initial rises in both the tibial and brachial artery waves. The estimation of the distance between baPWV sampling points was based on height. The formula (La-Lb)/Tba was used for calculating baPWV. Where La represents the distance between the brachium and the heart, and Tba represents the time interval between the ankle and brachial waveforms. An identical approach was used to measure baPWV for all patients, Right and left baPWV values were averaged for analytical purposes.

### Statistical analysis

Continuous data were presented as means ± standard deviations when normally distributed, or as medians (quartiles) when it was not distributed normally. Categorical variables were given as frequencies or percentages. These three data types were compared using one-way ANOVAs, Kruskal Wallis H tests, or chi-squared tests, respectively. The relationship between AIP and baPWV was assessed using univariate and multivariate linear regression models. Covariates were adjusted when, if added to the model, they altered the matched odds ratio by at least 10%. To detect non-linear associations, a generalized additive model (GAM) was also applied. When AIP/baPWV ratio was indicated in a smoothed curve, the inflection point was assessed by considering the recursive approach with a maximum likelihood model. Subgroup analyses were conducted with a stratified multivariate regression approach, and interaction analyses were presented in tabulated form.

EmpowerStats (http://www.empowerstats.com, X&Y Solutions, Inc, MA.USA) and R (http://www.R-project.org, The R Foundation) were applied for all statistical analyses. A two-sided P < 0.05 was the significance threshold.

## Results

### Baseline participant characteristics

In total, 4744 hypertensive patients were enrolled in this study (Fig. 1), including 2366 men (49.9%) and 2378 women (50.1%) between the ages of 29 and 93 (mean: 64.50 ± 9.40 years). The mean baseline AIP was -0.00 ± 0.28. Mean baPWV was 18.09 ± 3.88 m/s. The baseline characteristics of all the participants are shown in Table 1. For AIP quartiles, relative to patients with the highest AIP values (Q4) exhibited significantly higher BMI, waist circumference, heart rate, DBP, fasting blood glucose, total triglyceride, and serum uric acid levels compared with all other patients (Q1–Q3), whereas their age and HDL-c levels were significantly lower.

**Table 1:** Clinical characteristics of participants grouped by AIP quartiles Mean + SD/N(%)

Characteristics	Overall	Quartiles of AIP				
		Q1 (≥ − 0.87, < − 0.20)	Q2 (− 0.20, < − 0.01)	Q3 (≥ − 0.01, < 0.18)	Q4 (≥ 0.18, ≤ 0.94)	P − value
N	4744	1184	1187	1187	1186	
Age (year)	64.50 ± 9.40	66.89 ± 9.11	65.67 ± 9.16	63.75 ± 9.17	61.68 ± 9.32	< 0.001
Male (n%)	2366 (49.87%)	716 (60.47%)	576 (48.53%)	506(42.63%)	568(47.89%)	< 0.001
BMI (kg/m^2^)	23.25 ± 3.48	21.38 ± 3.09	22.72 ± 3.31	24.10 ± 3.40	24.81 ± 3.08	< 0.001
Waist circumference (cm)	82.13 ± 9.54	76.46 ± 8.89	80.76 ± 9.18	84.49 ± 8.81	86.80 ± 7.87	< 0.001
Heart rate (bpm)	75.67 ± 14.48	74.51 ± 15.59	74.92 ± 14.29	75.51 ± 13.31	77.73 ± 14.43	< 0.001
SBP (mmHg)	147.10 ± 17.55	147.45 ± 17.90	147.29 ± 17.63	147.15 ± 17.51	146.53 ± 17.14	0.600
DBP (mmHg)	88.79 ± 10.90	87.63 ± 11.10	88.21 ± 10.95	88.92 ± 10.62	90.40 ± 10.75	< 0.001
Fasting blood glucose; (mmol/L)	6.09 ± 1.55	5.82 ± 1.18	5.97 ± 1.57	6.11 ± 1.41	6.46 ± 1.88	< 0.001
Total cholesterol (mmol/L)	5.14 ± 1.10	5.01 ± 1.01	5.10 ± 1.11	5.29 ± 1.08	5.17 ± 1.17	< 0.001
Total triglyceride (mmol/L)	1.65 ± 0.92	0.83 ± 0.21	1.22 ± 0.26	1.68 ± 0.36	2.86 ± 0.92	< 0.001
HDL-c (mmol/L)	1.50 ± 0.39	1.86 ± 0.40	1.55 ± 0.30	1.40 ± 0.27	1.20 ± 0.26	< 0.001
LDL-c (mmol/L)	2.94 ± 0.79	2.57 ± 0.66	2.89 ± 0.77	3.15 ± 0.77	3.15 ± 0.80	< 0.001
AIP	− 0.00 ± 0.28	− 0.35 ± 0.11	− 0.11 ± 0.05	0.08 ± 0.06	0.37 ± 0.14	< 0.001
baPWV (m/s)	18.09 ± 3.88	18.19 ± 4.11	18.15. ± 4.04	18.07 ± 3.74	17.93 ± 3.59	0.374
eGFR (ml/min/1.73 m^2^)	86.23 ± 19.47	85.54 ± 19.23	84.75 ± 19.95	87.08 ± 18.50	87.53 ± 20.04	0.001
Serum uric acid (μmol/L)	430.57 ± 120.56	412.75 ± 117.50	415.11 ± 110.06	432.43 ± 120.35	461.97 ± 127.37	< 0.001
Homocysteine (μmol/L)	18.49 ± 11.62	18.57 ± 11.14	18.94 ± 12.13	18.19 ± 11.43	18.25 ± 11.75	0.004
Self-reported diabetes (n%)	373 (7.86%)	52 (4.39%)	74 (6.23%)	108 (9.10%)	139 (11.72%)	< 0.001
Coronary artery disease (n%)	292 (6.16%)	66 (5.57%)	78 (6.57%)	79 (6.66%)	69 (5.82%)	0.616
Stroke (n%)	305 (6.43%)	54 (4.56%)	87 (7.33%)	95 (8.00%)	69 (5.82%)	0.003
Current smoking (n%)	1351 (28.48%)	414 (34.97%)	347 (29.23%)	278 (23.42%)	312 (26.31%)	< 0.001
Current drinking (n%)	1178 (24.83%)	397 (33.53%)	278 (23.42%)	237 (19.97%)	266 (22.43%)	< 0.001
Labor intensity (n%)						< 0.001
Light	2570 (54.17%)	590 (49.83%)	646 (54.42%)	659 (55.52%)	675 (56.91%)	
Medium	1095 (23.08%)	277 (23.40%)	273 (23.00%)	259 (21.82%)	286 (24.11%)	
Heavy	1079 (22.74%)	317 (26.77%)	268 (22.58%)	269 (22.66%)	225 (18.97%)	
Antihypertensive drugs n (%)	2857 (60.22%)	648 (54.73%)	710 (59.81%)	757 (63.77%)	742 (62.56%)	< 0.001
Hypoglycemic drugs n (%)	187 (3.94%)	22 (1.86%)	39 (3.29%)	57 (4.80%)	69 (5.82%)	< 0.001
Antiplatelet drugs n (%)	104 (2.19%)	17 (1.44%)	32 (2.70%)	24 (2.02%)	31 (2.61%)	0.127

Values are presented as the mean ± SD or number (%)

AIP, the atherogenic index of plasma; BMI, body mass index; SBP, systolic blood pressure; DBP, diastolic blood pressure; baPWV, brachial-ankle pulse wave velocity; HDL-C, high-density lipoprotein cholesterol; LDL-C, low-density lipoprotein cholesterol; eGFR, estimated glomerular filtration rate

### The association between AIP and baPWV

**Fig. 2:**
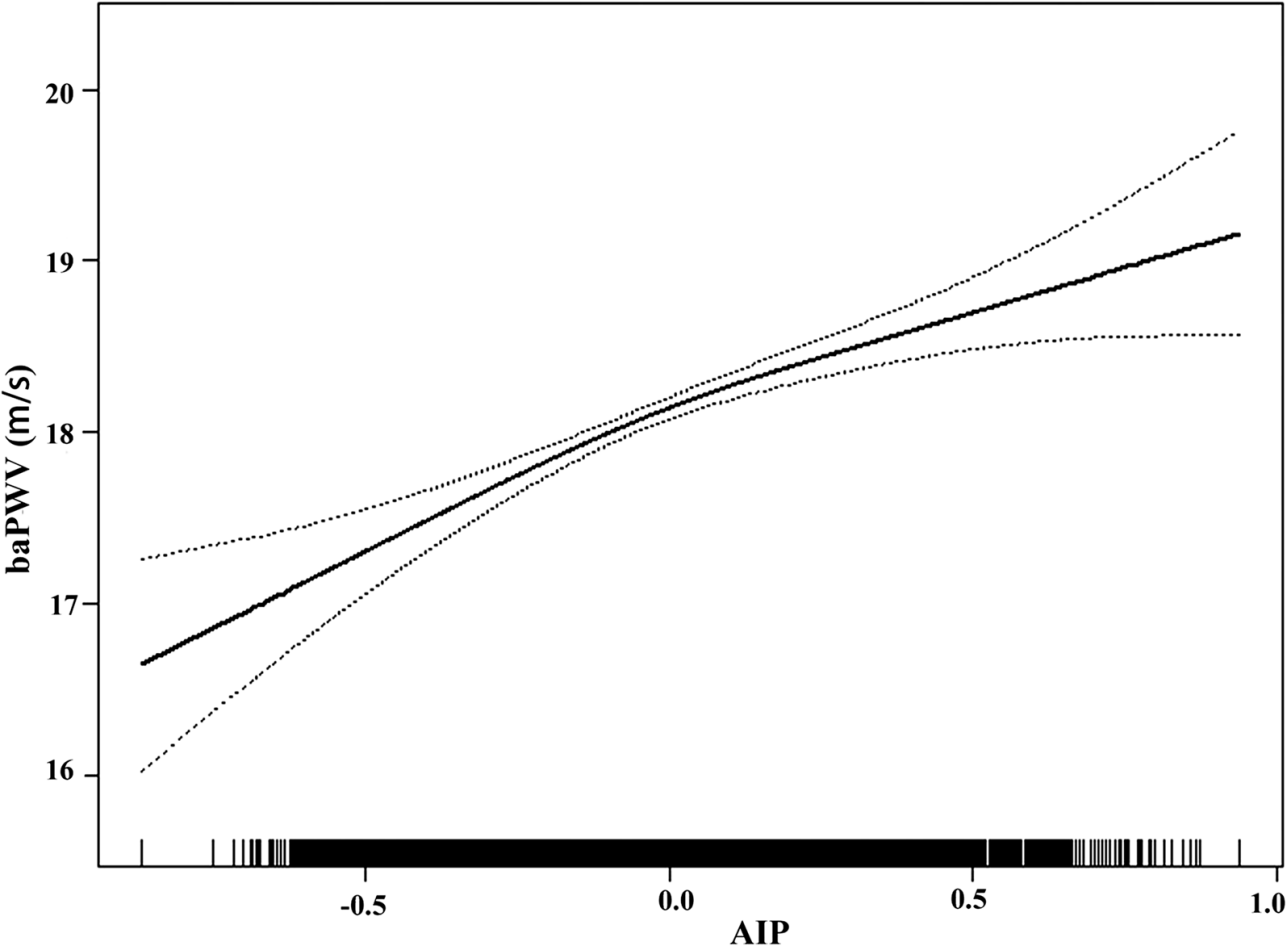
Smooth curve of correlation between AIP and baPWV. AIP, atherogenic index of plasma; baPWV, brachial-ankle pulse wave velocity. Smooth curve adjusted for age, sex, BMI, waist circumference, heart rate, SBP, DBP, fasting blood glucose, total cholesterol, LDL-L, eGFR, serum uric acid, homocysteine, self-reported diabetes, coronary artery disease, stoke, current smoking and drinking, labor intensity, antihypertensive drugs, hypoglycemic drugs, antiplatelet drugs

We utilized a multivariate linear regression model for assessing the association between AIP and baPWV (Fig. 2). As described in Table 2, in a minimally adjusted model rectified for patient age and sex, AIP was found positively correlated with baPWV (β = 1.07, 95% CI: 0.71 to 1.43, P < 0.001). After adjusting for age, sex, BMI, waist circumference, heart rate, SBP, DBP, fasting blood glucose, total cholesterol, LDL-c, eGFR, serum uric acid, homocysteine levels, self-reported diabetes, coronary artery disease, stroke, current smoking, current drinking, labor intensity, antihypertensive drugs, hypoglycemic drugs, and antiplatelet drugs (multivariate model II), AIP remained positively correlated with baPWV (β = 1.34, 95% CI: 0.96 to 1.72, P < 0.001). We additionally treated AIP as a categorical variable, separating patients based upon AIP quartiles, and again observed this same correlation (P for trend < 0.001).

**Table 2:** Relationship between AIP and baPWV in different modle

AIP baPWV	β (95%CI)	
	Model I	Model II
Per 1 unit increase	1.07 (0.71, 1.43)	1.34 (0.96, 1.72)
Quartiles		
Q1 (≥ − 0.87, < − 0.20)	Ref	Ref
Q2 (≥ − 0.20, < − 0.01)	0.13 (− 0.15, 0.41)	0.33 (0.08, 0.58)
Q3 (≥ − 0.01, < 0.18)	0.38 (0.10, 0.66)	0.70 (0.43, 0.97)
Q4 (≥ 0.18, ≤ 0.94)	0.66 (0.37, 0.94)	0.85 (0.56, 1.14)
P for trend	< 0.001	< 0.001

Model I: adjusted for age, sex

Model II: adjusted for age, sex, BMI, waist circumference, heart rate, SBP, DBP, fasting blood glucose, total cholesterol, LDL-L, eGFR, serum uric acid, homocysteine, Self-reported diabetes, coronary artery disease, stoke, current smoking, current drinking, labor intensity, antihypertensive drugs, hypoglycemic drugs, antiplatelet drugs

AIP, the atherogenic index of plasma; baPWV, brachial-ankle pulse wave velocity; β, beta coefficient; CI, confidence interval; Ref, reference

### Subgroup analysis results

Stratified and interaction analyses were next conducted for multivariate model II (Table 3). The results of testing for interactions were potentially important only for BMI (≥ 24 kg/m^2^ vs. < 24 kg/m^2^; P for interaction = 0.031), whereas these interactions did not display useful results for age (< 60 vs. ≥ 60 years; P for interaction = 0.845), sex (male vs. female; P for interaction = 0.108), SBP (< 140, 140–159, ≥ 160 mmHg; P for interaction = 0.995), DBP (< 90, 90–99, ≥ 100 mmHg; P for interaction = 0.740), eGFR (≥ 60 vs. < 60 ml/min**/**1.73m^2^; P for interaction = 0.646), homocysteine levels (≥ 15 vs. < 15 umol/l; P for interaction = 0.802), self-reported diabetes (no vs. yes; P for interaction = 0.949), coronary artery disease (no vs. yes;P for interaction = 0.106), stroke (no vs. yes; P for interaction = 0.616), antihypertensive drugs (no vs. yes; P for interaction = 0.087), current smoking (no vs. yes; P for interaction = 0.205), current drinking (no vs. yes; P for interaction = 0.739), or labor intensity (low, middle, high; P for interaction = 0.243). We found that there was a stronger correlation between AIP and baPWV in individuals with a BMI < 24 kg/m^2^ (β = 1.72, 95% CI: 1.17 to 2.28).

**Table 3:** Subgroup analyses of the effect size of AIP on baPWV

Characteristic	Number	β (95%CI)	P for interaction
Age (year)			0.845
< 60	1315	0.73 (0.23, 1.22)	
≥ 60	3429	1.02 (0.50 1.55)	
Sex (n %)			0.108
Male	2366	1.64 (1.12, 2.17)	
Female	2378	0.89 (0.35, 1.44)	
BMI (kg/m^2^)			0.031
< 24	2831	1.72 (1.17, 2.28)	
≥ 24	1911	0.89 (0.39, 1.39)	
SBP			0.995
< 140	1650	1.46 (0.90, 2.02)	
≥ 140, < 160	2032	1.49 (0.89, 2.08)	
≥ 160	1062	1.57 (0.54, 2.61)	
DBP			0.740
< 90	2491	1.48 (0.96, 2.01)	
≥ 90, < 100	1562	1.44 (0.78, 2.11)	
≥ 100	691	1.78 (0.61, 2.96)	
eGFR (ml/min/1.73 m^2^)			0.646
< 60	505	2.37 (0.91, 3.83)	
≥ 60	4239	1.29 (0.90, 1.67)	
HCY (μmol/L)			0.802
≤ 15	2303	1.16 (0.65, 1.67)	
> 15	2441	1.51 (0.95, 2.07)	
Self-reported diabetes (n %)			0.949
No	3920	1.30 (0.88, 1.72)	
Yes	824	1.53 (0.63, 2.43)	
Coronary artery disease (n %)			0.106
No	4452	1.31 (0.92, 1.69)	
Yes	292	1.99 (0.41, 3.57)	
Stroke (n %)			0.616
No	4439	1.32 (0.96, 1.74)	
Yes	305	1.77 (0.12, 3.22)	
Antihypertensive drugs n (%)			0.087
No	1887	1.39 (0.80, 1.97)	
Yes	2857	1.32 (0.82, 1.81)	
Current smoking (n %)			0.205
No	3393	1.00 (0.55, 1.46)	
Yes	1351	2.14 (1.47, 2.82)	
Current drinking (n %)			0.739
No	3566	1.26 (0.81, 1.70)	
Yes	1178	1.52 (0.81, 2.24)	
Labor intensity (n %)			0.243
Low	2570	1.35 (0.81, 1.89)	
Middle	1095	1.48 (0.79, 2.16)	
High	1079	1.32 (0.50, 2.13)	

Each group analysis adjusted, if not stratified, for age, sex, BMI, waist circumference, heart rate, SBP, DBP, fasting blood glucose, total cholesterol, LDL-L, eGFR, serum uric acid, homocysteine, self-reported diabetes, coronary artery disease, stoke, current smoking, current drinking, labor intensity, antihypertensive drugs, hypoglycemic drugs, antiplatelet drugs

AIP, the atherogenic index of plasma; baPWV, brachial-ankle pulse wave velocity; BMI, body mass index; SBP, systolic blood pressure; DBP, diastolic blood pressure; LDL-C, low-density lipoprotein cholesterol; eGFR, estimated glomerular filtration rate; β, beta coefficient; CI, confidence interval

## Discussion

The association of AIP with arterial stiffness has not been conclusively proven. The present study detected that AIP was positively correlated with baPWV in Chinese hypertensive patients. Several studies to date have assessed the relationship between single serum lipid parameters and arterial stiffness. Multiple prior studies having found elevated triglyceride levels and/or decreased HDL-C levels to be associated with an increase in such stiffness [26–28]. Additional, the majority of published papers found weak or no significant relationships between arterial stiffness and total cholesterol level or LDL-C level [29, 30]. Nevertheless, some studies showed inconsistent results [31, 32]. The use of two indices (triglyceride and HDL-C levels) to yield a single composite index (AIP) offers an effective approach to overcoming inconsistencies in the assessment of different lipid components. Therefore, This study aimed to clarify the association between comprehensive index of blood lipid (AIP) and arterial stiffness using baPWV in hypertensive patients from China.

Our findings were in line with data from a prior analysis of 249 normotensive and 366 untreated hypertensive individuals in Finland that found AIP to be associated with PWV [19]. Our research further observed the stability of this correlation in the subgroups of important covariates. In subgroup analysis, we found that BMI can change the relationship between AIP and baPWV. Si et al. [20] also reported AIP might be related to arterial stiffness in hypertensive patients, but this study had a limited sample size and failed to effectively adjust for potential confounding variables. Our study was a large-scale analysis of 4744 participants, thus can better use statistical adjustment to minimize the impact of potential confounding variables on our results, such as antihypertension medication, lipid-regulation medication and other confounding factors which were not adjusted by the previous study. We confirmed a positive linear correlation between AIP and baPWV in hypertensive patients from China. As such, as a representative of hypertensive Chinese individuals in the real-world, our study is the extension and supplement to the clinical study of AIP and arterial stiffness in Chinese population.

The AIP (log_10_[triglyceride/ HDL-C]) has recently been proposed as a superior index that better accounts for the interactions between different lipid fractions, reflecting the composition of plasma lipoproteins at the same time [33]. Relative to more traditional single lipid parameters, AIP exhibits a normal distribution and is thus better suited for the mathematical modeling of key cardiovascular variables [34]. Our data suggest that AIP values can offer information regarding patient arterial stiffness, indicating that this index can be reflected arterial stiffness in individuals suffering from essential hypertension. In primary community medical institutions where PWV testing cannot be widely carried out, especially in rural areas of China, the assessment methods for arteriosclerosis are still very limited. Being simple and fast, AIP is easy to be obtained from the information and calculation of the routine blood lipid tests without additional economic cost. Thus, the addition of AIP in routine blood lipid reports will have a broad application prospect in the assessment of arteriosclerosis in Chinese patients with hypertension.

The potential mechanisms regarding the relationship of the AIP with arterial stiffness are not entirely clear, The robust value of AIP in the assessment of cardiovascular disease risk is likely linked to its positive relationship with cholesterol esterification rates, lipoprotein particle size, and remnant lipoproteinaemia [35]. AIP reflects the distribution of small dense low-density lipoprotein (sdLDL) [36] and sdLDL levels are closely linked to oxidative stress and inflammation [37]. Many studies have confirmed oxidative stress and inflammation to contribute to arterial stiffness via aggravating endothelial dysfunction [38], promoting the upregulation of elastin-degrading enzymes [39], driving smooth muscle cell shifts from contractile to synthetic phenotypes [40] and enhancing fibroblast-derived extracellular matrix metallopeptidase expression [41]. These studies above may explain why AIP is positively correlated with arterial stiffness.

In subgroup analyses, We found that for individuals with a BMI < 24 kg/m^2^, AIP and baPWV were stronger correlated with one another. Some studies have reported that BMI or obesity is positively correlated with PWV or arterial stiffness [42, 43]. In a prospective study of 159 pubertal adolescents followed up for over 20 years and in a cross-sectional analysis of 336 subjects aged 36 years, Ferreira and colleagues found that the cfPWV increased with BMI [44]. A positive relationship between aortic stiffness and BMI in elderly participants was documented in Cardiovascular Health Study [45]. Zebekakis et al. also reported pulse wave velocity increased with higher BMI in middle-aged and older women [46]. Moreover, some studies also have shown that obesity or high BMI is associated with an increased risk of arterial stiffness. A random-effect meta-analysis study involving 15 case–control studies and 2237 children/adolescents (1281 obese patients and 956 healthy body mass index) found that obesity had a significant impact on PWV, and obesity was significantly associated with increased arterial stiffness [47]. The relationship between obesity and aortic stiffness was analyzed through a study of 186 youngsters and 177 elders. It is found higher BMI was closely related to higher PWV in both designated age groups, additionally, obesity and the obesity indicators were among the strongest independent predictors of pulse-wave velocity for both age groups [48]. We speculate that the effect of overweight or obesity on arterial stiffness is much greater than that of AIP on arterial stiffness, resulting in the weakened of the correlation between AIP and baPWV in overweight individuals. Therefore, in our subgroup analysis, there was a stronger positive association between AIP and baPWV in patients with BMI < 24 kg/m^2^.

This study has several limitations that must be considered. Firstly, this was a study of exclusively Chinese patients with essential hypertension, and thus, may not be relevant to other populations. Secondly, this was a cross-sectional analysis, and we were unable to draw any definitive conclusions with respect to the existence of any potential causal relationship between AIP and baPWV. Thirdly, we only used baPWV as an indicator of arterial stiffness. Although this indicator has been widely used in clinical and research studies, we still need more atherosclerosis tests to verify the relationship between AIP and arterial stiffness from all aspects in future studies, including the cardio-ankle vascular index (CAVI) and augmentation index (AIx), etc. Further, We need to have a reasonable design in the future and large-scale longitudinal studies to fully discover the potential association between AIP and arterial stiffness.

## Conclusion

In summary, we found that AIP was positively correlated with arterial stiffness in Chinese essential hypertension patients, and this relationship was a stronger correlation in those with a BMI < 24 kg/m^**2**^. These results indicate that AIP may represent a valuable surrogate predictor of arterial stiffness in Chinese hypertensive patients.

## Data Availability

The datasets used and analyzed during the current study are available from the corresponding author on reasonable request.

## Abbreviations

AIP: The atherogenic index of plasma
BMI: Body mass index
SBP: Systolic blood pressure
DBP: Diastolic blood pressure
baPWV: Brachial-ankle pulse wave velocity
HDL-C: High-density lipoprotein cholesterol
LDL-C: Low-density lipoprotein cholesterol
eGFR: Estimated glomerular filtration rate
β: Beta coefficient
CI: Confidence interval
Ref: Reference
